# Trends in antibiotic resistance among major bacterial pathogens isolated from blood cultures tested at a large private laboratory network in India, 2008–2014^☆^

**DOI:** 10.1016/j.ijid.2016.08.002

**Published:** 2016-09

**Authors:** Sumanth Gandra, Nestor Mojica, Eili Y. Klein, Ashvin Ashok, Vidya Nerurkar, Mamta Kumari, Uma Ramesh, Sunanda Dey, Viral Vadwai, Bibhu R. Das, Ramanan Laxminarayan

**Affiliations:** a Center for Disease Dynamics, Economics and Policy, New Delhi, India; b Johns Hopkins University, Department of Emergency Medicine, Baltimore, Maryland, USA; c Center for Disease Dynamics, Economics and Policy, 1400 Eye Street NW, Suite 500, Washington, DC, 20005, USA; d SRL Limited, Mumbai, India; e Public Health Foundation of India, Gurgaon, India; f Princeton Environmental Institute, Princeton, New Jersey, USA

**Keywords:** Antibacterial agents, Antibiotic resistance, Trends, India, Blood culture isolates

## Abstract

**Objective:**

There have been no long-term studies on trends in antibiotic resistance (ABR) on a national scale in India. Using a private laboratory network, the ABR patterns of organisms most commonly associated with bacteremia, obtained from patients across India between 2008 and 2014, were examined.

**Methods:**

A retrospective study of patient blood cultures collected over a 7-year period (January 1, 2008–December 31, 2014) was conducted. Data on the microorganism(s) identified and their antimicrobial susceptibility were obtained from SRL Diagnostics (Mumbai, India).

**Results:**

Of 135 268 blood cultures, 18 695 (14%) had at least one identified pathogen. In addition to continual high rates of methicillin-resistant *Staphylococcus aureus* (MRSA; approximately 44.2%), high resistance to nalidixic acid among Salmonella Typhi (98%) was observed, and carbapenem resistance increased in both *Escherichia coli* (7.8% to 11.5%; *p* = 0.332) and *Klebsiella pneumoniae* (41.5% to 56.6%; *p* < 0.001). Carbapenem resistance was also stable and high for both *Acinetobacter* species (approximately 69.6%) and *Pseudomonas aeruginosa* (approximately 49%). Resistance was also detected to colistin in the Gram-negatives and to vancomycin and linezolid in *S. aureus*.

**Conclusion:**

Increasing resistance to antibiotics of last-resort, particularly among Gram-negatives, suggests an urgent need for new antibiotics and improved antimicrobial stewardship programs in India.

## Highlights

• This study examined long-term trends in antibiotic resistance on a national scale in India.
• Colistin-resistant *Klebsiella pneumoniae* and *Escherichia coli* strains have emerged in India.
• In 2014, the prevalence of carbapenem-resistant *E. coli* was11.5%, the highest reported to date globally.

## 1 Introduction

Antibiotic resistance (ABR) is a major public health problem worldwide and is associated with adverse health and economic consequences.^1^ In India, a combination of mostly single-center studies and a few multicenter laboratory-based studies have shown a high prevalence of antibiotic resistance among common bacterial pathogens recovered from community- and hospital-acquired infections.^2^, ^3^, ^4^, ^5^, ^6^, ^7^ However, there have been no long-term studies on trends in ABR on a national scale in India.

Antimicrobial resistance surveillance on a national scale is critical because it provides information on the extent of established resistance rates, as well as emerging patterns of resistance. Understanding how resistance is changing is important for (1) establishing prescribing guidelines, (2) determining investment in new therapies, and (3) improving the targeting of campaigns to reduce antimicrobial resistance. It also provides a baseline for future analysis and comparison with other countries.

Rising rates of ABR in India are a significant concern because of the high burden of bacterial diseases^8^ and the poor health system infrastructure, which relies on antibiotics in place of adequate vaccination coverage and other public health measures. In this study, data from a large private laboratory network were used to examine the resistance patterns of the organisms most commonly associated with bacteremia in patients across India for the period January 2008 to December 2014.

## 2 Methods

**Figure 1:**
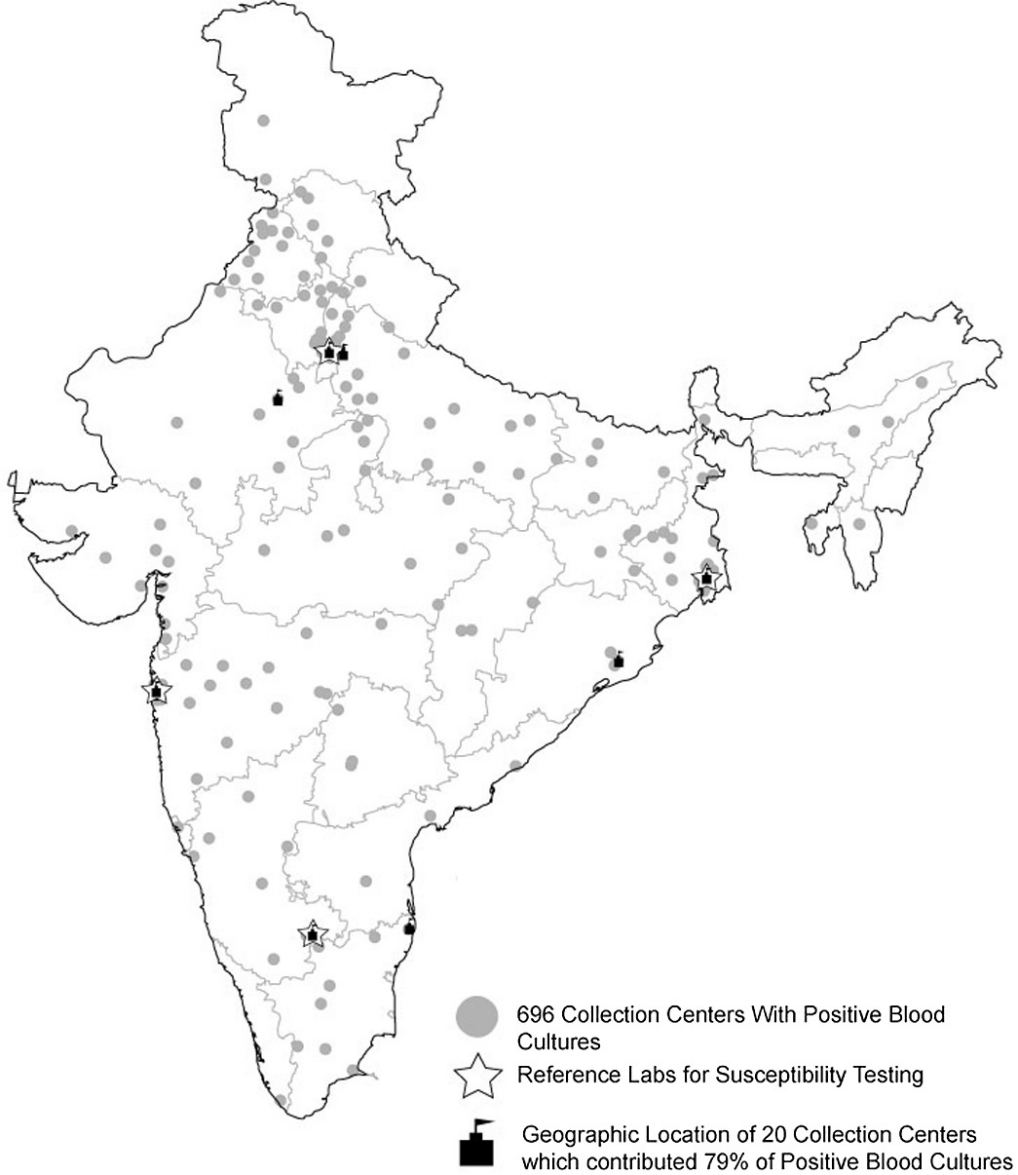
Geographic locations of the 696 collection centers with positive blood cultures.

This was a retrospective study of patient blood cultures collected over a 7-year period (2008–2014). Data on the microorganisms identified and their antimicrobial susceptibility were obtained from the SRL Limited laboratory network (Mumbai, India). The network includes 5700 collection centers spread across 26 of 29 states and two of seven Union Territories (UT). A collection center is a field site from where samples are collected based on physician orders. The collection centers include private hospitals (tertiary care, secondary care), diagnostic laboratories, and home collection agencies. Culture specimens collected are transported to the nearest of four reference laboratories located in four regions of the country (Figure 1) for isolation, organism identification, and antimicrobial susceptibility testing. The BACTEC 9050 (Becton Dickinson Microbiology Systems, Sparks, MD, USA) automated system was used to process blood cultures at all four reference laboratories.

Data were retrieved electronically from the actual patient reports. The following information was obtained: (1) final blood culture result (positive growth or no growth); (2) organism identified if the culture was positive; (3) interpreted susceptibility results for tested antimicrobials (susceptible, intermediate resistance, or resistant); (4) patient identifier and demographic information (age, sex); (5) collection center information (name and geographical location); and (6) date of specimen collection. Organism identification and antimicrobial susceptibility testing were performed using the broth microdilution methodology (MicroScan panels, Siemens, Sacramento, CA, USA) in all reference laboratories. Categorical result interpretations (susceptible, intermediate, and resistant) were based on up-to-date Clinical and Laboratory Standards Institute (CLSI) criteria at the time of testing.^9^ All culture-positive samples were considered without further interpretation of the results regarding clinical relevance.

The analysis considered all blood culture tests reported between January 1, 2008, and December 31, 2014. To avoid bias from duplicate cultures, data were filtered to retain only the first isolate from a patient. Identified microorganisms were stratified by year, age (<1, 1–17, 18–49, 50–64, and 65+ years), sex, and state or territory. Intermediate susceptible isolates were grouped with resistant isolates, as is now standard practice in the literature.^10^ Antimicrobial susceptibility results for major bacterial pathogens to clinically important antimicrobial agents were examined. The organisms examined were coagulase-negative staphylococci (CoNS), Salmonella Typhi and Paratyphi A, *Escherichia coli*, *Klebsiella pneumoniae*, *Acinetobacter* species, *Staphylococcus aureus*, *Pseudomonas aeruginosa*, *Enterococcus faecium*, and *Enterococcus faecalis.* Resistance was defined at the antibiotic class level using data from at least one of the several agents within the same class. For third-generation cephalosporins, susceptibility results were reported for ceftriaxone, cefotaxime, or ceftazidime for *Enterobacteriaceae*. For all organisms, carbapenem resistance was defined as intermediate resistance or resistance to meropenem or imipenem. Fluoroquinolone resistance for all organisms was defined as intermediate resistance or resistance to ciprofloxacin or levofloxacin, except for *Salmonella* species, where nalidixic acid was considered. Aminoglycoside resistance for all organisms was defined as intermediate resistance or resistance to gentamicin, tobramycin, or amikacin. As minimum inhibitory concentration (MIC) values were not available in the database, the resistance percentages for pathogen–antibiotic combinations in the years prior to the change in MIC breakpoints are not displayed.

### 2.1 Statistical analysis

Unadjusted resistance rates were calculated for each year as the number of resistant isolates as a proportion of total isolates tested. The Chi-square test (Cochran–Armitage) for linear trend was used to test the significance of annual trends. A *p*-value of ≤0.05 was considered statistically significant. All statistical analyses were performed using Stata software, version 12 (StataCorp, TX, USA). This study was approved by the Institutional Ethics Committee of the Public Health Foundation of India.

## 3 Results

### 3.1 Number and distribution of laboratories and cultures

**Figure 2:**
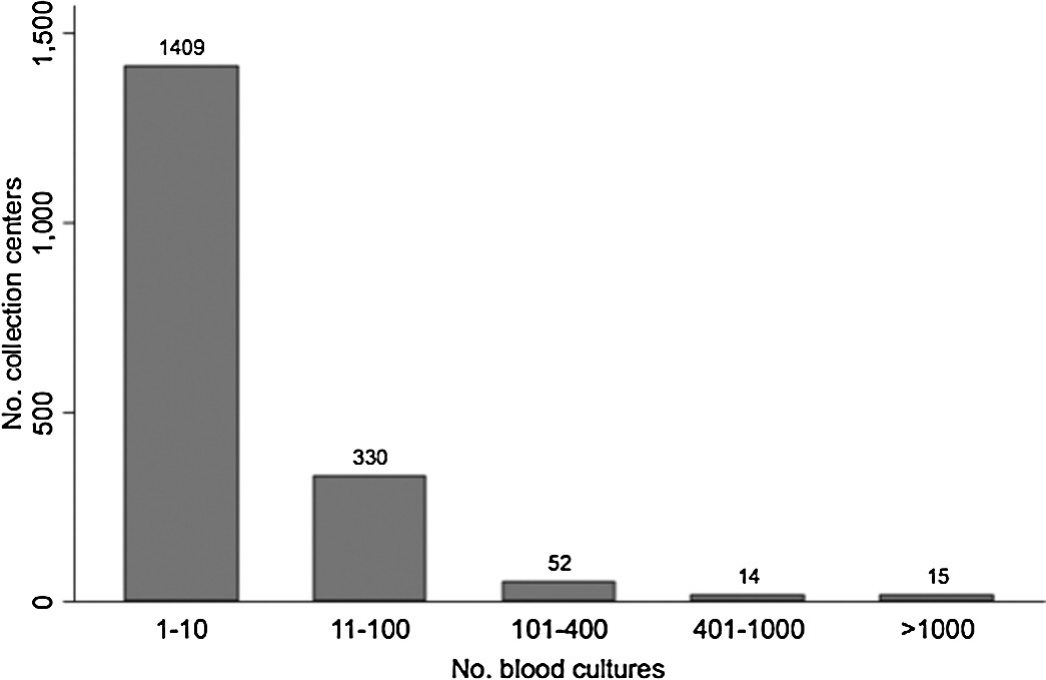
Blood culture contribution, by collection center.

A total of 135 268 blood cultures from unique persons were identified in the database for the period January 2008 to December 2014. Of these, 18 695 (14%) cultures were positive. Overall, the data came from 1820 unique collection centers spread across 425 cities and 27 states (including two UTs). The median number of blood cultures obtained from one collection center was 3 (interquartile range 1–99). Of the 1820 collection centers, 1409 (77.4%) contributed less than 11 blood cultures over 8 years (Figure 2). Positive blood cultures were identified at 696 of the centers spread across 185 cities, 25 states, and two UTs. The geographic distribution of collection centers contributing positive culture data is illustrated in Figure 1. Of the positive cultures, 79% were contributed by 20 collection centers that are tertiary care hospitals located in seven major cities (Figure 1), and 92.1% of positive cultures were from one UT (Delhi, 27.4%) and five states: Rajasthan (22.7%), Uttar Pradesh (21.2%), Maharashtra (11.0%), West Bengal (5.5%), and Karnataka (4.3%). Overall, Delhi UT had the highest contribution, with 34% of the total cultures and 27.4% of the positive cultures (Table 1). Approximately 62% of the total and positive cultures were from males and about 35% of the total cultures and 30% of the positive cultures were from persons aged 18–49 years (Table 1). Data on the distribution of total and positive cultures by year, age, sex, and state are given in the **Supplementary Material** (Tables S1 and S2, respectively).

### 3.2 Pathogen distribution

Of the 18 695 cultures that tested positive for at least one pathogen, 93.6% were bacteria and 6.4% were fungi. About 87.5% of pathogens were from one of 10 pathogen groups: CoNS (23.2%), *Salmonella* species (17.6%), *E. coli* (12.0%), *Klebsiella* species (7.9%), *S. aureus* (5.8%), *Candida* species (5.8%), *Acinetobacter* species (5.6%), *Pseudomonas* species (4.4%), *Enterococcus* species (2.8%), and *Enterobacter* species (2.5%). The remaining 12.5% of the identified pathogens included a wide variety of organisms (**Supplementary Material**, Table S3).

CoNS were the most common bacteria isolated in all years except 2008 and 2011 (Table 2). Among *Salmonella* species, 67% were Salmonella Typhi and 25% were Salmonella Paratyphi A. The database showed 66 polymicrobial cultures (cultures with more than one organism isolated). The three most common pathogens affecting infants (<1 year) were CoNS, *K. pneumoniae*, and *Candida* species. However, among pediatric individuals (aged 1–17 years) and young adults (aged 18–49 years), *Salmonella* species were the most common pathogens isolated. The three most common pathogens affecting older patients (>50 years) were CoNS, *E. coli*, and *K. pneumoniae*. More detailed information on the distribution of bacterial pathogens by age, sex, and state is given in the **Supplementary Material**(Table S4).

### 3.3 Antimicrobial susceptibility

**Figure 3:**
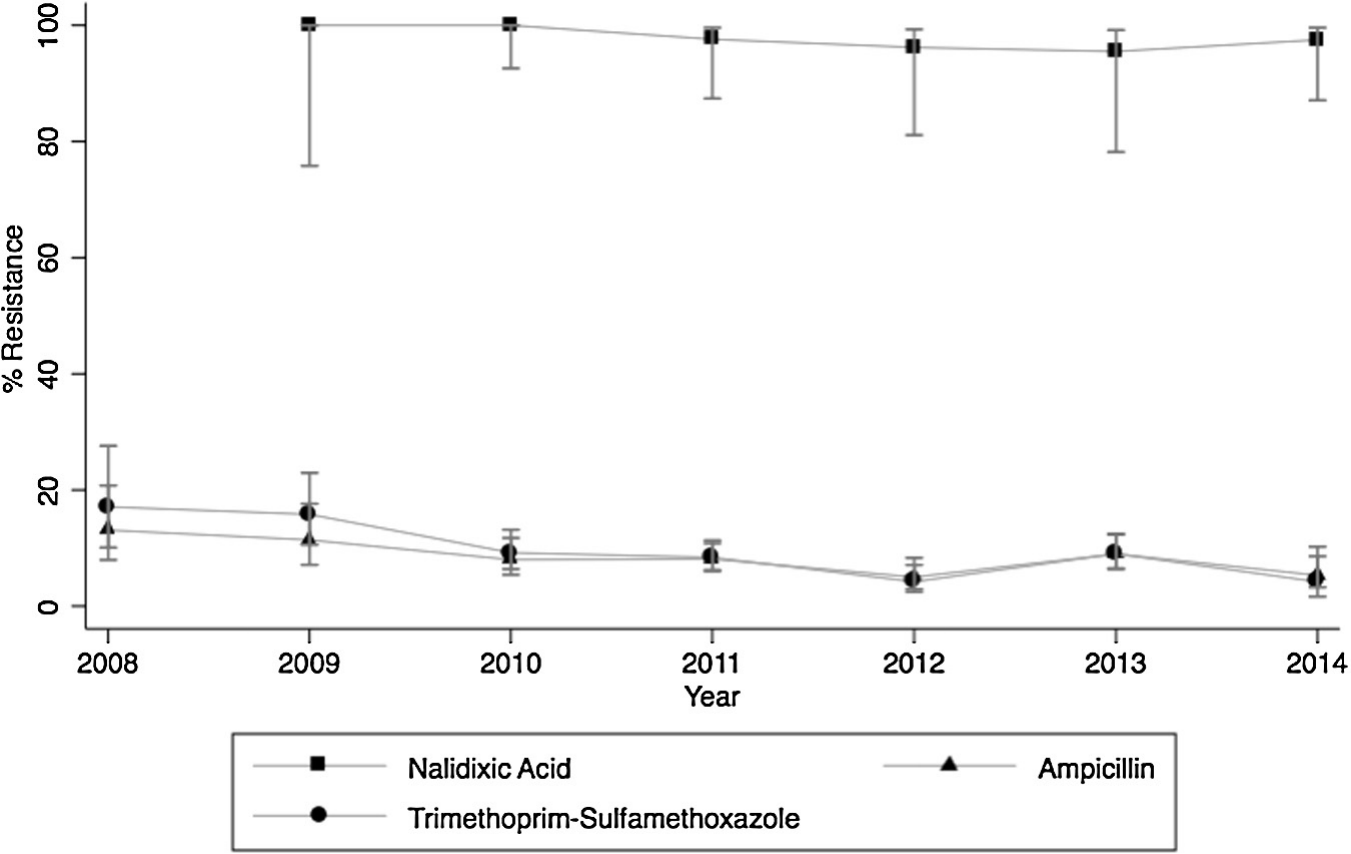
Resistance trends of Salmonella Typhi in India, 2008–2014 (error bars indicate the 95% confidence interval).

With regard to Gram-negative organisms, the average nalidixic acid resistance for all years among Salmonella Typhi was 98% (*n* = 190) and among Salmonella Paratyphi A was 96% (*n* = 67). Ampicillin and trimethoprim–sulfamethoxazole resistance among Salmonella Typhi decreased over the study period, dropping from 13.1% (*n* = 107) to 5.3% (*n* = 282) (*p* = 0.01) and from 17.1% (*n* = 70) to 4.2% (*n* = 96) (*p* < 0.001), respectively (Figure 3). Resistance to third-generation cephalosporins among *Salmonella* species was low, with 0.8% (*n* = 1841) of Salmonella Typhi and 1.1% (*n* = 657) of Salmonella Paratyphi A being resistant for all years (Table 3).

**Figure 4:**
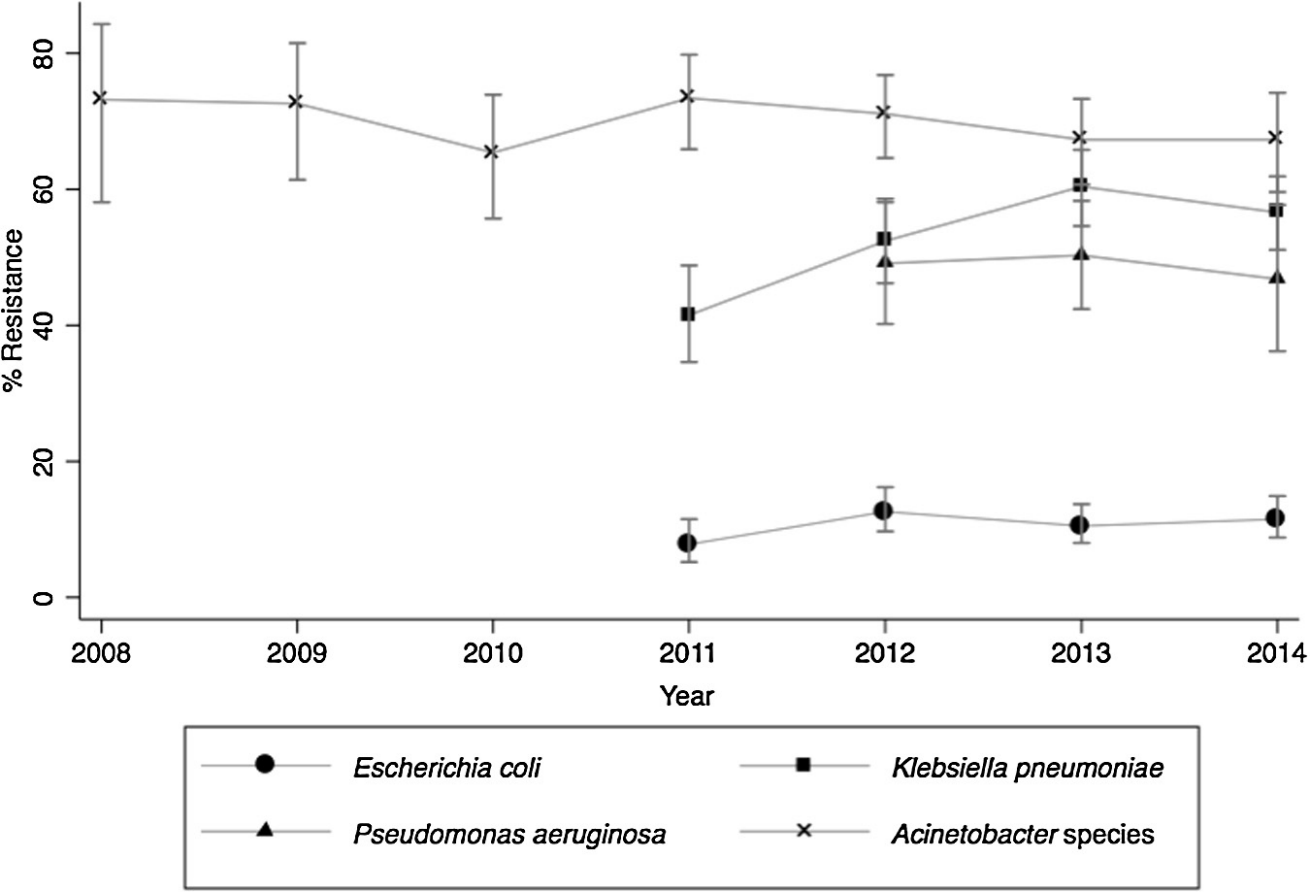
Carbapenem resistance trends among multiple organisms in India, 2008–2014 (error bars indicate the 95% confidence interval). For *E. coli* and *K. pneumoniae*, data are presented only from 2011, as the minimum inhibitory concentration (MIC) values were lowered in June 2010. For *P. aeruginosa*, data are presented from 2012, as MIC values were lowered in January 2012. For *Acinetobacter* species, MIC values were lowered in January 2014, and thus there is a possibility that resistance for the years 2008–2013 is underestimated.

Carbapenem resistance increased among *E. coli* (from 7.8% (*n* = 282) in 2011 to 11.5% (*n* = 426) in 2014; *p* = 0.332) and among *K. pneumoniae* (from 41.5% (*n* = 183) to 56.6% (*n* = 318); *p* < 0.001); however the increase was statistically significant only for *K. pneumoniae* (Figure 4). Among *Acinetobacter* species and *P. aeruginosa*, average carbapenem resistance was 69.6% (*n* = 994) for all years and 49% (*n* = 344) for the years 2012–2014, respectively, with no significant change in the trend observed for either organism during the study period (Figure 4). Colistin-resistant strains of *E. coli*, *K. pneumoniae*, *Acinetobacter* species, and *P. aeruginosa* were also detected as early as 2012, with resistance reaching 3.2% (*n* = 155) and 3.1% (*n* = 192) for *K. pneumoniae* and *E. coli* isolates, respectively, in 2014.

Among Gram-positive organisms, the average proportion of methicillin resistance and linezolid resistance in CoNS for all years was 73% (*n* = 2488) and 0.4% (*n* = 3579), respectively. Overall, three vancomycin-resistant CoNS isolates were observed during the study period (Table 3). Among *S. aureus*, the average proportion of methicillin-resistant *S. aureus* (MRSA) for all years was 44.2% (*n* = 608), with no significant change during the study period. Overall, two vancomycin-resistant *S. aureus* (VRSA), nine vancomycin-intermediate *S. aureus* (VISA), and 17 linezolid-resistant *S. aureus* (LRSA) isolates were reported during the study period. The average proportion of vancomycin resistance in *E. faecium* and *E. faecalis* for all years was 16.6% (*n* = 235) and 2.4% (*n* = 169), respectively, with no significant change during the study period.

## 4 Discussion

This study examined the ABR prevalence of bloodstream isolates obtained from patients across India. It greatly expands on prior studies of antimicrobial resistance in India,^2^, ^3^, ^4^, ^5^, ^6^, ^7^ providing detailed long-term descriptions of the profile and ABR patterns of organisms isolated in blood cultures from various regions of India. Overall, it was observed that Gram-negative organisms are frequently isolated in blood cultures. *Salmonella* species associated with enteric fever were the most frequently isolated Gram-negative organisms, followed by *E. coli* and *Klebsiella* species. Although CoNS represented the organisms most frequently isolated, this is likely because CoNS are a common contaminant in clinical specimens.^11^ This study provides evidence that enteric fever is the major cause of bacteremia primarily affecting children and young adults, justifying the need for improvements in sanitation and indicating the urgent need for a vaccine conferring long-term immunity. The low percentage of polymicrobial cultures (66 of 18 695 positive blood cultures) may indicate a low percentage of surgical patients in this database.

High resistance rates to both frontline antibiotics and those of last-resort were observed for all Gram-negative organisms isolated from blood cultures, but resistance was not static over the period of the study. For Salmonella Typhi and Salmonella Paratyphi A, nalidixic acid resistance rates remained extremely high (>95%), consistent with other studies performed in India,^3^, ^12^ while resistance rates to older antibiotics, ampicillin and trimethoprim–sulfamethoxazole, decreased over time. Similar findings were observed in single-center studies in India, with increasing susceptibility to older antibiotics like ampicillin, chloramphenicol, and trimethoprim–sulfamethoxazole.^13^, ^14^ These changes are likely due to the replacement of these drugs as an empiric treatment option for enteric fever with newer drugs, such as the fluoroquinolones. These findings suggest that fluoroquinolones can no longer be considered an empiric treatment option for suspected enteric fever; rather, physicians may be able to use older drugs again or third-generation cephalosporins (resistance to cephalosporins was minimal). Unfortunately, third-generation cephalosporin-resistant Salmonella Typhi and Salmonella Paratyphi A strains have emerged; these constituted about 0.8% of the isolates in this study, consistent with other studies in India.^3^, ^12^

The resistance rates of *E. coli* to fluoroquinolones and third-generation cephalosporins remained high throughout the study period; both were above 80% in 2014. High resistance rates were also observed for other antibiotics frequently used as empiric treatment options for *E. coli*, such as aminoglycosides (61.1%) and piperacillin–tazobactam (37.7%). Most alarming was the high carbapenem resistance, which was greater than 10%, a rate that is significantly higher than in other countries from which surveillance data are available. Of 41 countries with data from 2013 or 2014, only 12 reported detecting carbapenem resistance in *E. coli* and only three recorded a rate greater than 3%: Bulgaria (3.5%), Turkey (5%), and Vietnam (9%).^15^ Carbapenem resistance rates for *K. pneumoniae* also increased significantly over the study period, reaching approximately 60% by the end of the study, which again is higher than any other country except Greece, which had a similar percentage of carbapenem resistance in 2013 (60%).^16^ The rising carbapenem resistance among *E. coli* and *K. pneumoniae* isolates is a cause for concern, given the frequency of infections caused by these and the associated mortality, which for carbapenem-resistant *K. pneumoniae* bacteremia is estimated to be about 50%.^17^ The primary drug for treating carbapenem-resistant strains of *K. pneumoniae* and *E. coli* is colistin; however, worryingly, colistin resistance has already emerged. Colistin-resistant strains of *P. aeruginosa* and *Acinetobacter* species have also emerged. This is significant because these pathogens are intrinsically resistant to several antibiotics, leaving physicians with few options to treat infections.

Among Gram-positive organisms, *S. aureus* and *Enterococcus* species were the most frequently isolated organisms after CoNS. Methicillin resistance in CoNS was very high at 73%, consistent with other studies in India;^18^, ^19^, ^20^ however CoNS remain highly susceptible to vancomycin and linezolid. Similar to a multicenter study in India,^2^ the proportion of *S. aureus* isolates that were resistant to methicillin was high in this study (42% in the multicenter study vs. 44% in this study); however, of more significance were the isolates that were resistant to vancomycin and linezolid. Although *S. aureus* remains highly susceptible to both drugs, 3% of the isolates were linezolid-resistant in 2014. Several other studies in India have reported similar frequencies of LRSA, consistent with this finding.^21^, ^22^, ^23^ In the present study, two cases of VRSA were observed; however, vancomycin resistance in *Enterococcus* species was much higher, with 17% of the *E. faecium* isolates being vancomycin-resistant. Efforts have been made in the USA to reduce the threat posed by VRSA through reporting standards for infections caused by these pathogens.^24^ Similar efforts may be necessary in India, because genes conferring resistance, once they have evolved, can spread rapidly both within a country and around the world.

In this study, the resistance rates were also examined by age and sex. No significant differences were found (data not shown).

As with most large data surveillance studies, this study has limitations. First, although the analysis was confined to blood isolates, which likely portend infection, no clinical information was included. Second, information on variables differentiating community-acquired from healthcare-acquired infections was not available. However, enteric fever caused by *Salmonella* species (Salmonella Typhi and Paratyphi A) is a common cause of community-acquired bloodstream infection.^25^ Thus, it is likely that most if not all *Salmonella* cultures identified had a community origin. Third, although the data are national in scale, they may not be nationally representative of all Indian states or types of healthcare facility. Finally, MIC values were not available to re-interpret the resistance percentage for years prior to the change in MIC breakpoints. However, for the majority of pathogen and antibiotic combinations (28 of 37 combinations), the MIC values did not change during the study period.

In conclusion, increased antibiotic use has long been directly linked to higher rates of antibiotic resistance.^26^, ^27^ With the highest volume of antibiotic sales in 2010,^28^ it is not surprising that India has a simmering public health crisis related to antibiotic resistance. The increasing consumption of the two antibiotics of last-resort, carbapenems and polymyxins, between 2000 and 2010, portends a likely rise in the proportion of Gram-negative organisms resistant to these two antibiotic classes. As has been demonstrated before, resistance in India can spread rapidly to other parts of the world,^29^ making these results important not just for India. These results also indicate the urgent need for new antibiotics against Gram-negative organisms, as well as the necessity of continued surveillance of resistance patterns, especially in the Gram-negative organisms. Finally, the implementation of standard infection control practices and antimicrobial stewardship programs in healthcare facilities should be a priority.

*Funding:* This research received funding from the Bill & Melinda Gates Foundation (to CDDEP) for the Resistance Map project (SG, NM, RL). AA was supported by the Global Antibiotic Resistance Partnership, which is funded by the Bill & Melinda Gates Foundation. The funders had no role in the study design, in the collection, analysis, and interpretation of the data, in the writing of the report, or in the decision to submit the article for publication.

*Conflict of interest:* There are no conflicts of interest to disclose.

## Figures and Tables

**Table 1:** Distribution of total and positive blood cultures

	Total blood cultures	Positive cultures
	(*n* = 135 268)	(*n* = 18 695)
	Number (%)	Number (%)
Sex		
Male	83 055 (61.4)	11 561 (61.9)
Female	50 904 (37.6)	6904 (36.9)
Unknown	1309 (1.0)	220 (1.2)
Age, years		
<1	10 446 (7.7)	1814 (9.7)
1–17	19 595 (14.5)	2719 (14.5)
18–49	46 955 (34.7)	5601 (30.0)
50–65	28 661 (21.2)	4093 (21.9)
>65	29 246 (21.6)	4392 (23.5)
Unknown	365 (0.3)	76 (0.4)
Year		
2008	5381 (4.0)	695 (3.7)
2009	8553 (6.3)	1334 (7.1)
2010	14 731 (10.9)	2062 (11.0)
2011	21 709 (16.0)	3134 (16.8)
2012	29 676 (21.9)	3943 (21.1)
2013	29 706 (22.0)	3887 (20.8)
2014	25 512 (18.9)	3640 (19.5)
State		
Delhi	45 967 (34.0)	5128 (27.4)
Maharashtra	16 083 (11.9)	2061 (11.0)
Rajasthan	23 273 (17.2)	4245 (22.7)
Uttar Pradesh	24 777 (18.3)	3956 (21.2)
West Bengal	7328 (5.4)	1021 (5.5)
Karnataka	5557 (4.1)	799 (4.3)
Other	12 283 (9.1)	1485 (7.9)

**Table 2:** Ten most common organisms among positive cultures, 2008–2014; *n* (%)

Organism	2008	2009	2010	2011	2012	2013	2014	Total
CoNS	117 (16.8)	313 (23.4)	609 (29.5)	660 (21.1)	1031 (26.2)	792 (20.4)	815 (22.4)	4337 (23.2)
*Salmonella* species^a^	149 (21.4)	261 (19.5)	396 (19.2)	730 (23.2)	587 (14.9)	634 (16.3)	524 (14.4)	3281 (17.6)
*Escherichia coli*	66 (9.5)	166 (12.4)	202 (9.8)	312 (10.0)	501 (12.7)	495 (12.7)	496 (13.6)	2238 (12.0)
*Klebsiella* species	50 (7.2)	78 (5.9)	134 (6.5)	210 (6.7)	289 (7.3)	321 (8.3)	399 (11.0)	1481 (7.9)
*Staphylococcus aureus*	65 (9.4)	76 (5.7)	91 (4.4)	176 (5.6)	231 (5.9)	229 (5.9)	221 (6.1)	1089 (5.8)
*Candida* species	64 (9.2)	65 (4.9)	108 (5.2)	165 (5.3)	222 (5.6)	271 (7.0)	185 (5.1)	1080 (5.8)
*Acinetobacter* species	41 (5.9)	78 (5.9)	102 (5.0)	163 (5.2)	225 (5.7)	233 (6.0)	206 (5.7)	1048 (5.6)
*Pseudomonas* species	27 (3.9)	85 (6.4)	98 (4.8)	109 (3.5)	170 (4.3)	182 (4.7)	157 (4.2)	828 (4.4)
*Enterococcus* species	16 (2.3)	33 (2.5)	51 (2.5)	80 (2.6)	119 (3.0)	133 (3.4)	109 (3.0)	541 (2.9)
*Enterobacter* species	16 (2.3)	31 (2.3)	38 (1.8)	81 (2.6)	114 (2.9)	100 (2.6)	93 (2.5)	473 (2.5)
Other	84 (12.1)	148 (11.1)	233 (11.3)	448 (14.2)	454 (11.5)	497 (12.7)	435 (12.0)	2299 (12.3)
Number of isolates	695	1334	2062	3134	3943	3887	3640	18 695

CoNS, coagulase-negative staphylococci.

a Typhi and Paratyphi.

**Table 3:** Percentage of pathogenic isolates resistant (including intermediate isolates) to selected antibiotics, 2008–2014

Organism, antibiotics	2008	2009	2010	2011	2012	2013	2014	*p-*Value^b^
	Resistance, % (95% CI^a^)	Resistance, % (95% CI^a^)	Resistance, % (95% CI^a^)	Resistance, % (95% CI^a^)	Resistance, % (95% CI^a^)	Resistance, % (95% CI^a^)	Resistance, % (95% CI^a^)	
*Escherichia coli*								
Fluoroquinolones^c^	82.5 (71.4–90.0)	90.3 (84.8–93.9)	87.8 (82.4–91.6)	88.8 (84.8–91.8)	85.2 (81.8–88.1)	84.8 (81.2–87.7)	85.1 (81.4–88.1)	0.114
3rd-generation cephalosporins^d^	-g	-g	76.9 (70.5–82.3)	79.2 (74.0–83.5)	81.6 (77.8–84.8)	80.3 (76.4–83.8)	83.3 (79.4–86.5)	0.588
Carbapenems^e^	-g	-g	-g	7.8 (5.2–11.5)	12.6 (9.7–16.2)	10.5 (8.0–13.7)	11.5 (8.8–14.9)	0.332
Aminoglycosides^f^	61.3 (48.9–72.4)	74.6 (67.4–80.6)	70.4 (63.7–76.4)	66.8 (61.4–71.8)	70.1 (65.9–74.0)	63.2 (58.7–67.5)	61.1 (56.5–65.6)	0.003
Piperacillin–tazobactam	36.1 (25.2–48.7)	29.9 (23.4–37.3)	28.1 (22.2–34.7)	30.2 (25.3–35.6)	41.5 (37.2–46.0)	34.1 (29.8–38.6)	37.7 (33.2–42.3)	0.021
Colistin	-	-	-	0.0 (0.0–27.8)	3.4 (1.3–8.4)	1.1 (0.3–3.8)	3.1 (1.4–6.6)	0.785
*Klebsiella pneumoniae*								
Fluoroquinolones^c^	85.7 (73.3–92.9)	75.6 (65.1–83.8)	76.2 (68.1–82.7)	84.4 (78.8–88.7)	76.7 (71.4–81.2)	80.3 (75.4–84.4)	72.9 (67.8–77.4)	0.081
3rd-generation cephalosporins^d^	-g	-g	83.9 (76.6–89.2)	89.6 (84.6–93.1)	86.2 (81.7–89.8)	85.5 (81.0–89.0)	79.9 (75.2–83.9)	0.029
Carbapenems^e^	-g	-g	-g	41.5 (34.6–48.8)	52.4 (46.2–58.6)	60.4 (54.6–65.8)	56.6 (51.1–61.9)	<0.001
Aminoglycosides^f^	88.0 (76.2–94.4)	71.8 (61.0–80.6)	76.2 (68.1–82.7)	81.8 (75.9–86.5)	79.1 (74.0–83.4)	79.6 (74.7–83.8)	71.1 (66.0–75.8)	0.062
Piperacillin–tazobactam	54.5 (40.0–68.3)	50.6 (39.7–61.5)	58.9 (50.3–67.0)	67.7 (60.9–73.7)	65.9 (60.2–71.3)	68.1 (62.6–73.2)	62.7 (57.3–67.7)	0.052
Colistin	-	-	-	0.0 (0.0–48.9)	4 (1.6–9.9)	1.1 (0.3–3.8)	3.2 (1.4–7.3)	0.936
Salmonella Typhi								
Ampicillin	13.1 (8.0–20.8)	11.4 (7.1–17.6)	8.01 (5.4–11.7)	8.1 (6.0–10.8)	5.0 (2.9–8.3)	9.0 (6.5–12.3)	5.3 (3.2–8.6)	0.018
Fluoroquinolones^c^	100 (20.7–100)	100 (75.8–100)	100 (92.6–100)	97.6 (87.4–99.6)	96.2 (81.1–99.3)	95.5 (78.2–99.2)	97.5 (87.1–99.6)	0.269
Trimethoprim–sulfamethoxazole	17.1 (10.1–27.6)	15.8 (10.6–22.9)	9.2 (6.4–13.2)	8.4 (6.2–11.3)	4.2 (2.5–7.1)	9.0 (6.4–12.4)	4.2 (1.6–10.2)	<0.001
3rd-generation cephalosporins^d^	-g	-g	1.7 (1.0–4.0)	0.8 (0.3–2.0)	2.2 (1.1–4.3)	1.0 (0.4–2.7)	1.7 (0.7–3.9)	0.815
S/I/R^h^			282/3/2	512/3/1	350/6/2	377/0/4	294/0/5	
Salmonella Paratyphi A								
Ampicillin	4.2 (0.1–20.2)	4.5 (1.5–12.4)	2.1 (0.6–7.2)	3.1 (1.4–6.5)	2.5 (0.9–7.2)	1.5 (0.4–5.3)	2.7 (0.7–9.3)	0.366
Fluoroquinolones^c^	-	100 (72.2–100)	100 (80.6–100)	100 (78.5–100)	100 (51–100)	88.9 (56.5–98)	85.7 (60.1–96)	0.023
Trimethoprim–sulfamethoxazole	0.0 (0.0–22.8)	1.6 (0.3–8.7)	3.1 (1.1–8.7)	1.1 (0.3–3.9)	1.4 (0.4–5.1)	0.0 (0.0–3.2)	0.0 (0.0–12.8)	0.168
3rd-generation cephalosporins^d^	-g	-g	1.0 (0.2–5.6)	3.5 (1.7–7.0)	2.1 (0.7–6.0)	2.2 (1.0–6.3)	3.8 (1.3–10.6)	0.600
S/I/R^h^	-	-	96/1/0	195/6/1	140/2/1	132/1/2	76/0/3	

*Pseudomonas aeruginosa*								
Ceftazidime, cefepime	85.0 (63.9–94.8)	78.3 (67.2–86.4)	89.3 (80.9–94.3)	78.3 (68.3–85.8)	73.2 (64.7–80.2)	67.1 (59.3–74.1)	67.9 (57.3–76.9)	<0.001
Carbapenems^e^	-g	-g	-g	-g	49.1 (40.2–58.1)	50.3 (42.4–58.3)	46.8 (36.2–57.7)	0.792
Aminoglycosides^f^	75.0 (53.1–88.8)	58.0 (46.2–68.9)	71.8 (61.4–80.2)	65.1 (54.6–74.3)	53.7 (44.9–62.2)	57.2 (49.3–64.8)	56.6 (45.9–66.8)	0.044
Piperacillin–tazobactam	-g	-g	-g	-g	41.3 (33.0–50.2)	56.8 (48.7–64.6)	61.8 (50.6–71.9)	<0.001
Colistin	-	-	-	0.0 (0.0–65.8)	3.8 (1.0–12.8)	2.2 (0.6–7.7)	0.0 (0.0–7.6)	0.194

*Acinetobacter* species								
Carbapenems^e^	73.2 (58.1–84.3)	72.6 (61.4–81.5)	65.4 (55.7–73.9)	73.4 (65.9–79.8)	71.1 (64.6–76.8)	67.3 (60.7–73.3)	67.3 (59.6–74.2)	0.362
Colistin	-	-	-	-	2.5 (0.9–7.2)	5.9 (2.9–11.7)	4.1 (1.4–11.3)	0.435
CoNS								
Oxacillin	82.0 (69.2–90.2)	75.8 (69.8–80.8)	70.7 (66.8–74.4)	78.2 (73.5–82.3)	77.0 (73.2–80.3)	72.9 (68.7–76.8)	61.6 (56.3–66.7)	0.003
Vancomycin	0.0 (0.0–3.3)	0.0 (0–1.3)	0.0 (0.0–0.7)	0.0 (0.0–0.6)	0.0 (0.0–0.4)	0.0 (0.0–0.5)	0.6 (0.2–1.7)	0.011
S/I/R^h^	113/1/0	300/0/0	576/1/0	614/2/0	964/2/0	708/2/0	513/1/3	
Linezolid	0.0 (0.0–3.9)	0.0 (0.0–1.5)	0.0 (0.0–0.7)	0.0 (0.0–0.7)	0.0 (0.0–0.4)	0.1 (0.0–0.8)	2.5 (1.5–4.1)	<0.001
*Staphylococcus aureus*								
Oxacillin	50.0 (30.7–69.3)	28.6 (18.9–40.7)	48.7 (37.8–59.7)	40.7 (30.9–51.3)	53.1 (44.5–61.4)	40.0 (32.0–48.6)	46.5 (37.1–56.2)	0.342
Vancomycin	0.0 (0.0–6.2)	0.0 (0.0–5.7)	1.4 (0.2–7.4)	0.6 (0.1–3.6)	2.4 (1.1–5.6)	0.0 (0.0–2.0)	2.2 (0.9–5.6)	0.218
S/I/R^h^	58/0/0	63/0/0	72/0/1	152/1/0	199/5/0	184/0/0	176/4/0	
Linezolid	13.3 (6.3–26.2)	0.0 (0.0–6.3)	0.0 (0.0–5.1)	0.0 (0.0–2.6)	2.6 (1.1–6.0)	0.5 (0.0–3.0)	3.3 (1.5–7.0)	0.198
*Enterococcus faecium*								
Ampicillin	75.0 (30.1–95.4)	100 (70.1–100)	83.9 (67.4–92.9)	80.9 (67.5–92.9)	92.7 (82.7–97.1)	92.2 (83.0–96.6)	97.1 (85.5–99.5)	0.040
Vancomycin	0.0 (0.0–43.4)	0.0 (0.0–27.8)	32.3 (18.6–49.9)	17.8 (9.3–31.3)	18.5 (10.4–30.8)	16.4 (9.4–27.1)	10.5 (4.2–24.1)	0.459
*Enterococcus faecalis*								
Ampicillin	-	14.3 (2.6–51.3)	13.3 (3.7–37.9)	3.7 (0.7–18.3)	13.2 (5.8–27.3)	11.6 (5.1–24.5)	16.2 (7.7–31.1)	0.487
Vancomycin	-	0.0 (0.0–32.4)	5.9 (1.0–27.0)	4.0 (0.7–19.5)	0.0 (0.0–9.4)	0.0 (0.0–7.7)	5.6 (1.5–18.1)	0.095

CoNS, coagulase-negative staphylococci.

a Wilson 95% confidence interval.

b *p-*Values obtained using the Cochran–Armitage test.

c Fluoroquinolones include ciprofloxacin and levofloxacin (nalidixic acid for Salmonella Typhi and Paratyphi A).

d Third-generation cephalosporins include ceftriaxone, cefotaxime, and ceftazidime.

e Carbapenems include imipenem and meropenem.

f Aminoglycosides include gentamicin, tobramycin, and amikacin.

g Resistance percentages not displayed, as minimum inhibitory concentration (MIC) values changed during the study period.

h S, susceptible; I, intermediate; R, resistant.
