# Supplementary material for: Trends in antibiotic resistance among major bacterial pathogens isolated from blood cultures tested at a large private laboratory network in India, 2008–2014
Source: Int J Infect Dis. 2016 Sep;50:75–82. doi: 10.1016/j.ijid.2016.08.002 (PMC5063511; doi:10.1016/j.ijid.2016.08.002)
Supplement: Supplementary file 1 [file mmc1.docx]

| **Supplementary Table S1:** Demographic Distribution of Total Blood Cultures | | | | | | | |
| --- | --- | --- | --- | --- | --- | --- | --- |
|  | **2008** | **2009** | **2010** | **2011** | **2012** | **2013** | **2014** |
|  | No. (%) | No. (%) | No. (%) | No. (%) | No. (%) | No. (%) | No. (%) |
| Gender |  |  |  |  |  |  |  |
| Male | 3,374 (62.7) | 5,255 (61.5) | 9,212 (62.5) | 13,442 (61.9) | 18,004 (60.7) | 18,283 (61.6) | 15,485 (60.7) |
| Female | 1,929 (35.9) | 3,177 (37.2) | 5,315 (36.1) | 8,069 (37.2) | 11,432 (38.5) | 11,189 (37.7) | 9,793 (38.4) |
| Unknown | 78 (1.45) | 121 (1.4) | 204 (1.4) | 197 (0.9) | 240 (0.8) | 304 (0.8) | 234 (0.9) |
|  |  |  |  |  |  |  |  |
| Age |  |  |  |  |  |  |  |
| <1 | 454 (8.4) | 747 (8.7) | 1,241 (8.4) | 1,352 (6.2) | 2,678 (9.0) | 1,983 (6.7) | 1,991 (7.8) |
| 1-17 | 821(15.3) | 1575 (18.4) | 2,466 (16.7) | 2,974 (13.7) | 4,083 (13.8) | 4,146 (14.0) | 3,530 (13.8) |
| 18-49 | 2,092(38.9) | 3073 (35.9) | 5,594 (38.0) | 7.955 (36.6) | 10,007 (33.7) | 10,185 (34.3) | 8,049 (31.6) |
| 50-65 | 982(18.3) | 1610 (18.8) | 2,823 (19.2) | 4,600 (21.2) | 6,252 (21.1) | 6,386 (21.5) | 6,008 (23.6) |
| >65 | 1,032(19.2) | 1548 (18.1) | 2,606 (17.7) | 4,820 (22.2) | 6,567 (22.1) | 6,760 (22.8) | 5,913 (23.2) |
| Unknown | -- | -- | 1 (0.01) | 8 (0.04) | 89 (.03) | 246 (0.8) | 21 (0.08) |
|  |  |  |  |  |  |  |  |
| States |  |  |  |  |  |  |  |
| Delhi | 1,574 (29.3) | 2646 (30.9) | 5,041 (34.2) | 8.143 (37.5) | 9,279 (31.3) | 11269 (38.0) | 8,015 (31.4) |
| Rajasthan | 1,316 (24.5) | 1557 (18.4) | 2,279 (15.5) | 2,974 (13.7) | 5,884 (19.8) | 4,686 (15.8) | 4,557 (17.9) |
| Uttar Pradesh | 1,359 (25.3) | 2372 (27.7) | 3,933 (26.7) | 4,342 (20.0) | 4,522 (15.2) | 4,231 (14.2) | 4,018 (15.8) |
| Maharashtra | 600 (11.15) | 847 (9.9) | 1,335 (9.1) | 2,453 (11.3) | 4,730 (15.9) | 3,402 (11.5) | 2,716 (10.7) |
| Karnataka | 52 (1.0) | 256 (3.0) | 607 (4.1) | 643 (3.0) | 1395 (4.7) | 1196 (4.0) | 1408 (5.5) |
| West Bengal | 210 (3.9) | 313 (3.7) | 814 (5.5) | 1,236 (5.7) | 1,884 (6.4) | 2,023 (6.8) | 848 (3.3) |
| Other | 270 (5.0) | 542 (6.3) | 722 (4.9) | 1918 (8.8) | 1982 (6.7) | 2899 (9.8) | 3950 (15.5) |
|  |  |  |  |  |  |  |  |
| Total | 5,381 | 8,553 | 14,731 | 21,709 | 29,676 | 29,706 | 25,512 |

| **Supplementary Table S2:** Demographic Distribution of Positive Blood Cultures | | | | | | | |  |
| --- | --- | --- | --- | --- | --- | --- | --- | --- |
|  | **2008** | **2009** | **2010** | **2011** | **2012** | **2013** | **2014** | |
|  | No. (%) | No. (%) | No. (%) | No. (%) | No. (%) | No. (%) | No. (%) | |
| Gender |  |  |  |  |  |  |  | |
| Male | 432 (62.2) | 836 (62.7) | 1,331 (64.6) | 1,919 (61.2) | 2,453 (62.2) | 2,402 (61.8) | 2,198 (60.4) | |
| Female | 246 (35.4) | 475 (35.6) | 690 (33.5) | 1,181 (37.7) | 1,467 (37.2) | 1,453 (37.4) | 1,392 (38.2) | |
| Unknown | 17 (2.45) | 23 (1.7) | 41 (2.0) | 34 (1.1) | 23 (0.6) | 32 (0.8) | 50 (1.4) | |
|  |  |  |  |  |  |  |  | |
| Age |  |  |  |  |  |  |  | |
| <1 | 85 (12.2) | 177 (13.3) | 255 (12.4) | 237 (7.6) | 420 (10.7) | 300 (7.7) | 340 (9.3) | |
| 1-17 | 131(18.9) | 242 (18.1) | 350 (17.0) | 504 (16.1) | 497 (12.6) | 499 (12.9) | 496 (13.6) | |
| 18-49 | 228 (32.8) | 393 (29.5) | 675 (32.7) | 1,026 (32.6) | 1174 (29.8) | 1,119 (28.8) | 986 (27.1) | |
| 50-65 | 124 (17.8) | 260 (19.5) | 415 (20.1) | 635 (20.3) | 858 (21.8) | 890 (22.9) | 911 (25.0) | |
| >65 | 127 (18.3) | 262 (19.6) | 367 (17.8) | 730 (23.3) | 969 (24.6) | 1,035 (26.6) | 902 (24.8) | |
| Unknown | -- | -- | -- | 2 (0.1) | 25 (0.6) | 44 (1.1) | 5 (0.1) | |
| States |  |  |  |  |  |  |  | |
|  |  |  |  |  |  |  |  | |
| Delhi | 151 (21.7) | 371 (27.8) | 600 (29.1) | 1,022 (32.6) | 925 (23.5) | 1,132 (29.1) | 927 (25.5) | |
| Rajasthan | 256 (36.8) | 316 (23.7) | 413 (20.0) | 523 (16.7) | 1,091 (27.7) | 855 (22.0) | 791 (21.7) | |
| Uttar Pradesh | 147 (21.2) | 328 (24.6) | 576 (27.9) | 776 (24.8) | 773 (19.6) | 695 (17.9) | 661 (18.1) | |
| Maharashtra | 63 (9.1) | 115 (8.6) | 154 (7.5) | 346 (11.0) | 571 (14.5) | 433 (11.1) | 379 (10.4) | |
| Karnataka | 8 (1.2) | 25 (1.9) | 59 (2.9) | 93 (3.0) | 220 (5.6) | 195 (5.0) | 199 (5.5) | |
| West Bengal | 35 (5.0) | 108 (8.1) | 166 (8.1) | 167 (5.33) | 144 (3.7) | 286 (7.4) | 115 (3.2) | |
| Other | 35 (5.0) | 71 (5.3) | 94 (4.6) | 207 (6.6) | 219 (5.6) | 291 (7.5) | 568 (15.6) | |
|  |  |  |  |  |  |  |  | |
| Total | 695 | 1,334 | 2,062 | 3,134 | 3,943 | 3,887 | 3,640 | |

| **Supplementary Table S3:** ‘Other’ Organisms | | |
| --- | --- | --- |
| Organisms | No. | % |
| *Streptococcus species* | 430 | 18.7 |
| *Aerobic spore species* | 317 | 13.79 |
| *Burkholderia species* | 202 | 8.79 |
| *Micrococcus species* | 172 | 7.48 |
| *Stenotrophomonas species* | 154 | 6.7 |
| *Contaminants* | 133 | 5.79 |
| *Serratia species* | 91 | 3.96 |
| *Chryseobacterium species* | 83 | 3.61 |
| *Yeast cells* | 83 | 3.61 |
| *Citrobacter species* | 67 | 2.91 |
| *Proteus species* | 58 | 2.52 |
| *Diptheroids* | 57 | 2.48 |
| *Achromobacter species* | 56 | 2.44 |
| *Gram Positive bacilli organism* | 54 | 2.35 |
| *Gram Negative bacilli organism* | 50 | 2.17 |
| *Empedobacter* | 40 | 1.74 |
| *Morganella species* | 33 | 1.44 |
| *Ralstonia species* | 32 | 1.39 |
| *Aeromonas species* | 17 | 0.74 |
| *Yersinia species* | 17 | 0.74 |
| *Providencia species* | 16 | 0.7 |
| *Aerococcus species* | 10 | 0.43 |
| *Cryptococcus species* | 10 | 0.43 |
| *Trichosporon species* | 9 | 0.39 |
| *Haemophilus parainfluenzae* | 7 | 0.3 |
| *Ochrobactrum species* | 7 | 0.3 |
| *Alcaligenes species* | 6 | 0.26 |
| *Kluyvera species* | 6 | 0.26 |
| *Sphingomonas species* | 6 | 0.26 |
| *Tatumella species* | 6 | 0.26 |
| *Elizabethkingia species* | 5 | 0.22 |
| *Leuconostoc species* | 4 | 0.17 |
| *Rhodotorula species* | 4 | 0.17 |
| *Acremonium species* | 3 | 0.13 |
| *Cedecea species* | 3 | 0.13 |
| *Chromobacteriums species* | 3 | 0.13 |
| *Escherichia species* | 3 | 0.13 |
| *Pantoea species* | 3 | 0.13 |
| *Pasteurella species* | 3 | 0.13 |
| *Rothia species* | 3 | 0.13 |
| *Shigella species* | 3 | 0.13 |

| **Supplementary Table S3** (*Continued)* |  |  |
| --- | --- | --- |
| Organisms | No. | % |
| *Staphylococcus species* | 3 | 0.13 |
| *Bordetella species* | 2 | 0.09 |
| *Delftia species* | 2 | 0.09 |
| *Gamella species* | 2 | 0.09 |
| *Hafinia alvei* | 2 | 0.09 |
| *Legionella pneumophila* | 2 | 0.09 |
| *Myroides species* | 2 | 0.09 |
| *Prototheca species* | 2 | 0.09 |
| *Raoultella species* | 2 | 0.09 |
| *Rhizobium species* | 2 | 0.09 |
| *Clostridium species* | 1 | 0.04 |
| *Edwardsiella tarda* | 1 | 0.04 |
| *Flavobacterium species* | 1 | 0.04 |
| *Fusarium species* | 1 | 0.04 |
| *Geotrichum species* | 1 | 0.04 |
| *Gliocladium* | 1 | 0.04 |
| *Kocuria species* | 1 | 0.04 |
| *Kodamaea species* | 1 | 0.04 |
| *Listeria monocytogenes* | 1 | 0.04 |
| *Moraxella species* | 1 | 0.04 |
| *Neisseria species* | 1 | 0.04 |
| *Rhodococcus species* | 1 | 0.04 |
| Total | 2,299 |  |

| **Supplementary Table S4:** Demographic Distribution of Selected Pathogens | | | | | | | | |
| --- | --- | --- | --- | --- | --- | --- | --- | --- |
|  |  |  |  |  |  |  |  |  |
|  | coagulase negative *staphylococcus* | *Escherichia coli* | *Klebsiella pnuemoniae* | *Salmonella species (*Typhi/Paratyphi*)* | *Pseudomonas aeruginosa* | *Acinetobacter species* | *Staphylococcus aureus* | *Candida species* |
|  | No. (%) | No. (%) | No. (%) | No. (%) | No. (%) | No. (%) | No. (%) | No. (%) |
| Gender |  |  |  |  |  |  |  |  |
| Male | 2722 (62.8) | 1189 (53.1) | 934 (63.1) | 1983 (60.4) | 537 (64.9) | 692 (66.0) | 753 (69.1) | 692 (64.1) |
| Female | 1584 (36.5) | 1039 (46.4) | 535 (36.1) | 1271 (38.7) | 281 (33.9) | 348 (33.2) | 320 (29.4) | 368 (34.1) |
| Unknown | 30 (0.7) | 10 (0.5) | 12 (0.8) | 27 (0.8) | 10 (1.2) | 8 (0.8) | 16 (1.5) | 20 (1.8) |
|  |  |  |  |  |  |  |  |  |
| Age |  |  |  |  |  |  |  |  |
| <1 | 554 (12.8) | 86 (3.8) | 245 (16.5) | 58 (1.8) | 53 (6.4) | 84 (8.0) | 89 (8.2) | 205 (19.0) |
| 1-17 | 493 (11.4) | 38 (1.7) | 59 (4.0) | 1455 (44.4) | 59 (7.1) | 91 (8.7) | 103 (9.5) | 31 (2.9) |
| 18-49 | 1123 (25.9) | 389 (17.4) | 394 (26.6) | 1661 (50.6) | 246 (29.7) | 344 (32.8) | 314 (28.8) | 269 (24.9) |
| 50-65 | 991 (22.9) | 744 (33.2) | 380 (25.7) | 87 (2.7) | 241 (29.1) | 269 (25.7) | 313 (28.7) | 276 (25.6) |
| >65 | 1148 (26.5) | 981 (43.8) | 396 (26.7) | 19 (0.6) | 226 (27.3) | 255 (24.3) | 269 (24.7) | 288 (26.7) |
| Unknown | 27 (0.62) | -- | 7 (0.5) | 1 (0.03) | 3 (0.4) | 5 (0.5) | 1 (0.1) | 11 (1.0) |
|  |  |  |  |  |  |  |  |  |
| States |  |  |  |  |  |  |  |  |
| Delhi | 742 (17.1) | 868 (38.8) | 313 (21.1) | 1568 (47.8) | 178 (21.0) | 239 (22.8) | 291 (26.7) | 97 (9.0) |
| Rajasthan | 1212 (27.9) | 246 (11.0) | 328 (22.1) | 587 (17.9) | 192 (23.2) | 177 (16.9) | 228 (20.9) | 430 (39.8) |
| Uttar Pradesh | 1304 (30.1) | 375 (16.8) | 305 (20.6) | 304 (9.3) | 185 (22.3) | 296 (28.2) | 218 (20.0) | 237 (21.9) |
| Maharashtra | 419 (9.7) | 225 (10.0) | 201 (13.6) | 354 (10.8) | 118 (14.2) | 174 (16.6) | 93 (8.5) | 174 (16.1) |
| Karnataka | 230 (5.3) | 121 (5.4) | 31 (2.1) | 181 (5.5) | 30 (3.6) | 26 (2.5) | 36 (3.3) | 30 (2.8) |
| West Bengal | 208 (4.8) | 184 (8.2) | 153 (10.3) | 88 (2.7) | 48 (5.8) | 65 (6.2) | 65 (6.0) | 38 (3.5) |
| Other | 221 (5.1) | 219 (9.8) | 150 (10.1) | 199 (6.1) | 77 (9.3) | 71 (6.8) | 158 (14.5) | 74 (6.8) |
|  |  |  |  |  |  |  |  |  |
